# Elastomeric respirators: Expanding the “E” in PPE

**DOI:** 10.1017/ice.2020.1377

**Published:** 2020-12-15

**Authors:** Michael P. Koster, Leonard A. Mermel

**Affiliations:** Department of Infection Control and Hospital Epidemiology, Alpert Medical School of Brown University, Rhode Island Hospital and Hasbro Children’s Hospital of Lifespan, 593 Eddy St, Providence, RI 02903

*To the Editor*—The Centers for Disease Control and Prevention (CDC) recently acknowledged that transmission severe acute respiratory coronavirus virus 2 (SARS-CoV-2) may include small-particle aerosols over greater distances, necessitating institutions to seek more appropriate respiratory protection for their patient-facing staff.^1^ Elastomeric respirators are an alternative to provide the needed level of protection that N95 respirators currently provide. Unlike N95 respirators that are traditionally single use, elastomeric respirators are intended for extended use, and with appropriate cleaning processes, for reuse.^2^ Given the increase in cases of coronavirus disease 2019 (COVID-19) across the United States and the fractured supply chains for personal protective equipment such as N95 respirators, we performed an informal survey of academic institutions to determine whether their staff are currently using elastomeric respirators when caring for patients with COVID-19 infection.

We surveyed hospital epidemiologists from 45 institutions via e-mail, and we received 9 responses (~20% response rate) across 7 states: Colorado, Massachusetts, Maryland, North Carolina, Rhode Island, Utah, and Virginia. All 9 institutions invested in elastomeric respirators for their hospital employees, some specifically for certain departments (ie, respiratory therapy, anesthesiology, otolaryngology, and general surgery). A few had already been using elastomeric respirators in operating rooms before the COVID-19 pandemic, with initial use after the 2009 H1N1 pandemic. Due to asymptomatic spread and need for source control in the COVID-19 pandemic, all of these institutions reported that they require a surgical mask be placed over the exhalation valve of the elastomeric respirator. Notably, not unique to elastomeric respirators, users described communication interference.^3^ Still, we believe that elastomeric respirators are an additional tool many institutions are utilizing and others should be exploring. The next step at our institution will be rolling out elastomeric respirators, fit-testing them, and requiring that the exhalation valve be covered with a surgical mask while awaiting newly designed valve covers from the manufacturer.
